# The Suitability of Glioblastoma Cell Lines as Models for Primary Glioblastoma Cell Metabolism

**DOI:** 10.3390/cancers12123722

**Published:** 2020-12-11

**Authors:** Anya L. Arthurs, Damien J. Keating, Brett W. Stringer, Simon J. Conn

**Affiliations:** Flinders Health and Medical Research Institute, Flinders University, Adelaide, SA 5042, Australia; damien.keating@flinders.edu.au (D.J.K.); brett.stringer@flinders.edu.au (B.W.S.); simon.conn@flinders.edu.au (S.J.C.)

**Keywords:** metabolism, glioblastoma, oncology, cell culture, metabolic flux, glycolysis

## Abstract

**Simple Summary:**

Glioblastoma (GBM) is a deadly brain tumour with no effective treatments. Recently, new treatments which target the cancer’s unique metabolic properties are beginning to emerge. However, this preclinical research is commonly undertaken in human cell lines which poorly recapitulate the properties of the cancer in situ. This study has examined the metabolic properties of five commonly used GBM cell lines in comparison to healthy brain and GBM tissue. While no cell line faithfully recapitulates GBM, certain lines are useful for aspects of metabolic analysis in GBM cells. We identified three cell lines which accurately reflect the mitochondrial metabolism of GBM tumours, and one cell line suited for studies into glycolysis. In addition to providing detailed metabolic profiles of these commonly used cell lines, this research can guide preclinical experiments to assess the efficacy of desperately needed, novel therapeutics for GBM.

**Abstract:**

In contrast to most non-malignant tissue, cells comprising the brain tumour glioblastoma (GBM) preferentially utilise glycolysis for metabolism via “the Warburg effect”. Research into therapeutics targeting the disease’s highly glycolytic state offer a promising avenue to improve patient survival. These studies often employ GBM cell lines for in vitro studies which translate poorly to the in vivo patient context. The metabolic traits of five of the most used GBM cell lines were assessed and compared to primary GBM and matched, healthy brain tissue. In patient-derived GBM cell lines, the basal mitochondrial rate (*p* = 0.043) and ATP-linked respiration (*p* < 0.001) were lower than primary adjacent normal cells from the same patient, while reserve capacity (*p* = 0.037) and Krebs cycle capacity (*p* = 0.002) were higher. Three cell lines, U251MG, U373MG and D54, replicate the mitochondrial metabolism of primary GBM cells. Surprisingly, glycolytic capacity is not different between healthy and GBM tissue. The T98G cell line recapitulated glycolysis-related metabolic parameters of the primary GBM cells and is recommended for research relating to glycolysis. These findings can guide preclinical research into the development of novel therapeutics targeting metabolic pathways in GBM.

## 1. Introduction

Glioblastoma (GBM) is an aggressive brain tumour with a poor survival rate (5 year survival rate < 10%) which has improved only 1% in the past 30 years [1]. Despite being the most common malignant brain tumour in adults, successful therapies for GBM have not been found, resulting in a median survival time of only 12–15 months, post-diagnosis [2]. Within the last decade, research has turned to examining the therapeutic potential of drugs interrupting the metabolism of GBM cells, leading to slower proliferation rates and/or reduced metastasis [3]. Like many cancers, GBM preferentially uses the less-efficient glycolytic pathway to produce energy in the form of adenosine 5′-triphosphate (ATP) and nicotinamide adenine dinucleotide hydride (NADH) rather than the oxidative phosphorylation pathway favoured by most somatic cells. This shift in metabolic pathways is called “the Warburg effect” and results in the accumulation of lactate regardless of oxygen availability [4]. Dichloroacetate, which targets a key glycolytic pathway enzyme pyruvate dehydrogenase kinase [5,6], has been demonstrated to inhibit tumour progression in vivo in lung and breast cancer [5,7].

GBM tumours exhibit heterogeneity, both of the cells they comprise and the cellular metabolism profiles [8], which complicates their profiling. Often primary human GBM cells are unavailable, or instead xenografted in the murine brain to allow genetic manipulation, in vivo [9]. This can confound metabolic analyses compared with the primary tumour due to the effects from the tumour microenvironment [10]. As a result, many researchers utilise established GBM cell lines as in vitro models. While different metabolic profiles have been completed for both primary GBM cells and GBM cell lines in vitro, there has been no standardised, comprehensive comparison. This consolidated research is necessary as in vitro culturing conditions are known to effect cell growth and thus cellular metabolism [11,12,13].

This diversity in modelling the GBM disease presents a challenge, as research into potential therapies which target metabolism may be confounded by which cell lines or models are used during the preclinical phase. To address this knowledge gap, we have compared the metabolic profiles of primary GBM cells (derived from GBM tumours) with primary healthy normal mixed neural cells from the same patients, as well as five of the most commonly used human GBM cell lines: U87MG, U251MG, U373MG, D54 and T98G. This provided a reliable comparison of primary GBM cell metabolism with cell lines used for research, as well as with normal neural cells, to provide a clearer understanding of which GBM cell lines most closely represent primary GBM metabolism.

## 2. Results

### 2.1. Baseline Oxygen Consumption Rate (OCR) Comparison of Primary Healthy and GBM Cells, and GBM Cell Lines

**Figure 1:**
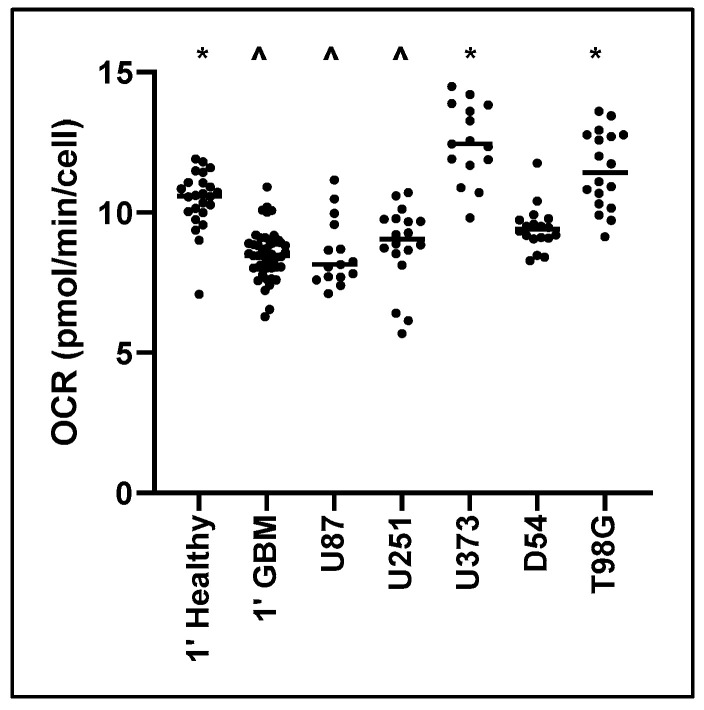
Baseline Oxygen Consumption Rate (OCR) for primary healthy cells and primary Glioblastoma (GBM) cells, and GBM cell lines. Cell populations of primary healthy mixed neural cells, primary GBM cells, and GBM cell lines, were isolated and mitochondrial metabolic activity was measured as changes in OCR. Data presented represent the mean and individual data points. Biological replicates for cell lines (*n* = 5), primary healthy cells (*n* = 8), and primary GBM tumour cells (*n* = 13); with technical triplicates. * Significant difference from primary glioblastoma control (1′ GBM) (*p* ≤ 0.05). ^ Significant difference from primary healthy mixed neural control (1′ Healthy) (*p* ≤ 0.05).

Baseline Oxygen Consumption Rate (OCR) was measured to determine the normal mitochondrial respiration of different cells and cell lines. No significant variation was observed specific to patient sex or nationality. Primary healthy mixed neural cells exhibited significantly higher OCR (27%) compared to primary GBM cells from the same patient (*p* < 0.001) (Figure 1). Furthermore, U373MG (*p* < 0.001) and T98G (*p* < 0.001) displayed significantly higher baseline OCR compared with only the primary GBM tumour control. U87MG and U251MG were only significantly decreased compared with the primary healthy control (U87MG *p* < 0.001, U251MG *p* = 0.011). This indicates that most cell lines display different mitochondrial metabolism in culture compared with primary resected tissue.

### 2.2. Comparison of Mitochondrial Function in Response to Metabolism-Altering Compounds

**Figure 2:**
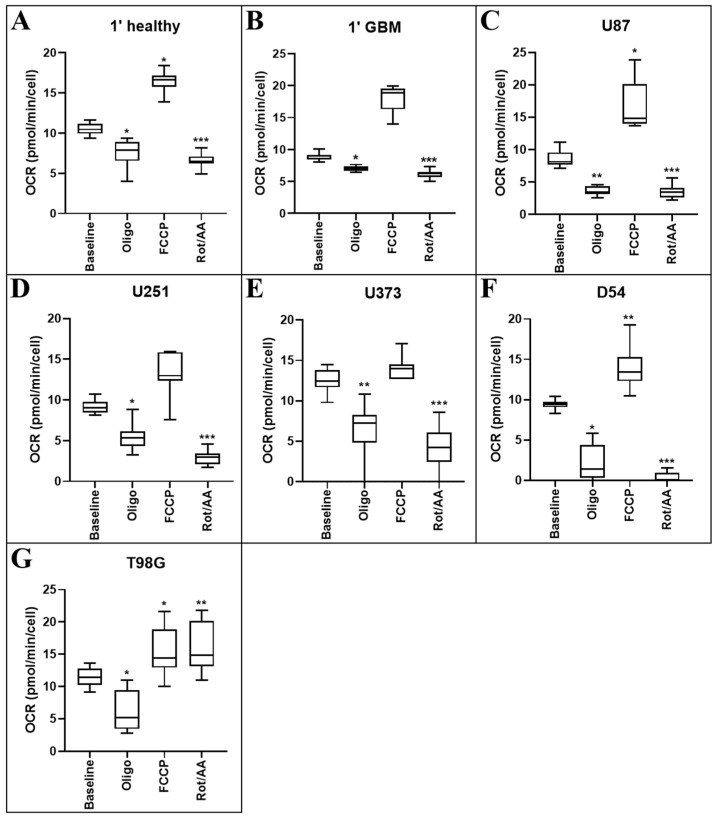
Mitochondrial respiration rate in primary healthy and GBM cells, and GBM cell lines. Cell populations of (**A**) primary mixed neural cells, (**B**) primary GBM cells, (**C**) U87MG, (**D**) U251MG, (**E**) U373MG, (**F**) D54 and (**G**) T98G cells were isolated and mitochondrial metabolic activity was measured as changes in OCR. Data presented represent the mean, with quartile 1 and 3 marked. For all data points in cell lines, *n* = 5; in primary healthy cells, *n* = 8; in primary GBM cells, *n* = 13; plated in triplicate. * above error bars show a statistically significant comparison between baseline control and treatment group. * *p* ≤ 0.05, ** *p* ≤ 0.01, and *** *p* ≤ 0.001.

Oligomycin is the first compound injected into the Seahorse plate wells. Oligomycin inhibits the proton channel of ATP synthase in cells, hindering the cell’s ability to produce ATP and consume oxygen via mitochondrial oxidative phosphorylation [14]. As such, OCR is expected to decrease upon exposure to this compound. All cells responded in this manner, showing significantly reduced OCR when treated with oligomycin (healthy *p* = 0.027; 1′ GBM *p* = 0.048; U87MG *p* = 0.004; U251MG *p* = 0.007; U373MG *p* < 0.001; D54 *p* = 0.029; T98G *p* = 0.002) when compared with their respective baselines (Figure 2).

Following oligomycin treatment, carbonyl cyanide-4-phenylhydrazone (FCCP) is injected. Treatment with the protonophore FCCP, a potent uncoupler of mitochondrial oxidative phosphorylation, disrupts ATP synthesis by interfering with the proton gradient generated by the electron transport chain before it can be utilised for energy. A significant increase in OCR is expected following administration of this compound. Healthy cells (*p* = 0.044), U87MG (*p* = 0.042), D54 (*p* = 0.024) and T98G (*p* = 0.019) cells showed significantly increased OCR following FCCP administration when compared with their respective baselines, with the remaining lines being unaffected (Figure 2).

Finally, a mix of rotenone and antimycin A is injected into the wells. This inhibits mitochondrial electron transport complexes I and III, respectively. This is expected to significantly decrease the OCR of cells following FCCP addition. All cells had significantly reduced OCR when treated with rotenone/antimycin A (healthy *p* < 0.001; 1′ GBM *p* < 0.001; U87MG *p* < 0.001; U251MG *p* < 0.001; U373MG *p* < 0.001; D54 *p* < 0.001) when compared with their respective baselines, except the T98G cell line which was unaffected with this treatment.

### 2.3. Metabolic Parameters Pertaining to OCR and the Extracellular Acidification Rate (ECAR)

**Figure 3:**
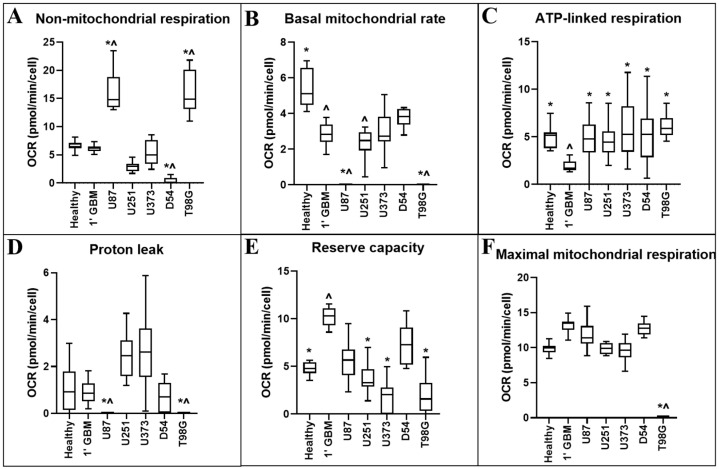
Comparison of OCR-related metabolic parameters of primary neural and GBM cells, and GBM cell lines. OCR-related metabolic parameters: (**A**) non-mitochondrial respiration; (**B**) the basal mitochondrial rate; (**C**) ATP-linked respiration; (**D**) proton leak; (**E**) reserve capacity; and (**F**) maximal mitochondrial respiration were calculated from measurements obtained using information from the Seahorse Bioscience Metabolic Flux Analyser Mito Stress Kit. Data presented represent the mean, with quartile 1 and 3 marked. For all data points in cell lines, *n* = 5; in primary healthy cells, *n* = 8; in primary GBM cells, *n* = 13; plated in triplicate. * represents statistically significant difference with primary glioblastoma control (1′ GBM) (*p* ≤ 0.05). ^ represents statistically significant difference with between primary healthy mixed neural control (1′ Healthy) (*p* ≤ 0.05).

The addition of metabolism-altering compounds oligomycin, FCCP and rotenone/antimycin A allows for calculation of metabolic parameters pertaining to the OCR and the Extracellular Acidification Rate (ECAR) of the cells. Table 1 and Figure 3 display the statistical and diagrammatic results, respectively, of metabolic parameters pertaining to OCR.

Non-mitochondrial respiration records the consumption of oxygen occurring in alternative locations of the cell to the mitochondrion, such as at the cell surface via transplasma membrane electron transport, or by cytosolic enzymes such as nicotinamide adenine dinucleotide phosphate hydrogen (NADPH) oxidase [15].

The basal mitochondrial rate provides a representation of the baseline mitochondrial respiration of the cells in standard assay conditions.

The ATP-linked respiration rate (oxygen consumption driven by ATP synthesis in mitochondria) provides a representation of several parameters, including ATP demand or substrate supply and oxidation in the cell.

Mitochondrial proton leak describes the diffusion of protons across the inner membrane independently of ATP synthase, and results in continued mitochondrial oxygen consumption in the presence of oligomycin. Proton leak can be affected by several factors including the uncoupling protein family or mitochondrial membrane damage.

The reserve capacity rate, or spare respiratory capacity, provides a representation of the cell’s ability to meet increased energy demands and changes can reflect alterations in mitochondrial biogenesis/mass or altered arrangement of oxidative phosphorylation components on the inner mitochondrial membrane.

The maximal respiration rate provides a representation of the greatest amount of respiration the cell can provide whilst FCCP uncouples the proton gradient in the electron transport chain. This measurement relies on several parameters, including mitochondrial biogenesis or substrate supply and oxygen transport/metabolism in the cell.

**Figure 4:**
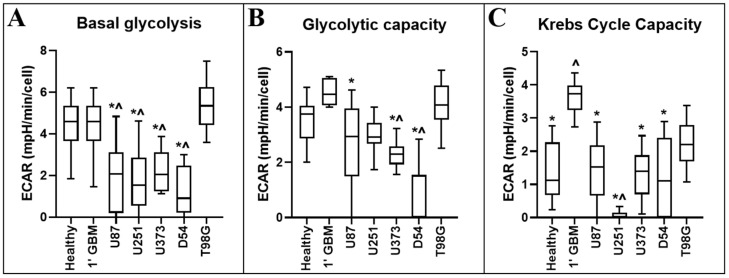
Comparison of ECAR-related metabolic parameters of primary neural and GBM cells, and GBM cell lines. ECAR-related metabolic parameters: (**A**) basal glycolysis (the basal ECAR); (**B**) glycolytic capacity (the ECAR after oligomycin); and (**C**) Krebs cycle capacity were calculated from measurements obtained using information from the Seahorse Bioscience Metabolic Flux Analyser Mito Stress Kit. Data presented represent the mean, with quartile 1 and 3 marked. For all data points in cell lines, *n* = 5; in primary healthy cells, *n* = 8; in primary GBM cells, *n* = 13; plated in triplicate. * represents statistically significant difference with primary glioblastoma control (1′ GBM) (*p* ≤ 0.05). ^ represents statistically significant difference with between primary healthy mixed neural control (1′ Healthy) (*p* ≤ 0.05).

Glycolysis is determined through measurements of the ECAR of the surrounding media, which is predominantly from the excretion of lactic acid after its conversion from pyruvate [14]. Table 2 and Figure 4 display the statistical and diagrammatic results, respectively, of metabolic parameters pertaining to the ECAR.

Oligomycin inhibits the proton channel of ATP synthase in cells, hindering their ability to produce ATP via mitochondrial oxidative phosphorylation, requiring cells to increase glycolytic flux as a compensatory mechanism to meet cellular ATP demands [14]. Thus, the ECAR following oligomycin treatment provides an indication of the maximal glycolytic capacity of the cell population of interest.

FCCP has been shown to often increase the ECAR in cells. This increase is primarily caused by increased CO_2_ production from the Krebs cycle, upregulated to meet demand for NADH and flavin adenine dinucleotide (FADH_2_) from the electron transport chain (ETC). Carbon dioxide converts to bicarbonate and protons in an aqueous environment, thus contributing to extracellular acidification [14]. The measurement of the ECAR difference from FCCP treatment to oligomycin treatment is taken as a measure of CO_2_ production and acidification of the medium. As oligomycin almost entirely inhibits mitochondrial oxygen consumption, Krebs cycle activity is likely stalled, as cofactors NADH/FADH_2_ are no longer required for the ETC. This measurement is therefore largely reflective of glycolysis/glycolytic flux. When FCCP treatment is added, maximal rate of the ETC is initiated and the cell ineffectually attempts to reinstate mitochondrial proton gradient (which is then dissipated by FCCP). However, this sudden maximal ETC activity requires cofactors NADH/FADH_2_ as electron donors and Krebs cycle activity is increased to meet the high demand. In addition to these products, Krebs cycle also produces an amount of CO_2_, thus acidifying its aqueous medium environment.

## 3. Discussion

We have shown that a panel of the most widely used GBM model cell lines fail to faithfully recapitulate the metabolic activity of primary GBM tumour cells. Furthermore, we have specifically identified several metabolic parameters in which primary GBM cells differ from primary healthy mixed neural cell populations, as well as those parameters which are best mirrored in GBM cell lines. Importantly, the majority of commonly used GBM cell lines (excepting T98G) had a significantly decreased baseline glycolysis compared to primary healthy cells, reaffirming the need to target the correct cell lines for bespoke research projects.

Metabolism in the brain relies on an interaction between neurons and astrocytes. Glycogen is predominantly found in astrocytes [16] which contain enzymes to activate high levels of glycolytic metabolism, mainly releasing lactate [17,18]. Lactate and pyruvate transfer can occur to localised neurons [19], where this enters the Krebs cycle and can continue through the oxidative phosphorylation pathway. Thus, an interplay between astrocytes and neurons is required for successful metabolic activity, underscoring our choice of a mixed neural cell population for our primary healthy cells.

Some metabolic parameters of cell activity were found to be similar between primary healthy and GBM cells. As summarized in Table 3, primary healthy and GBM cells were not statistically different in their non-mitochondrial respiration rates or maximal mitochondrial respiration rates, nor were they different in their basal ECAR or the ECAR after oligomycin. It is important to note that a lack of significant difference in glycolytic capacity between primary healthy and GBM cells does not indicate that the GBM cells are not utilizing the Warburg effect, as the GBM cells are preferentially utilizing glycolysis over oxidative phosphorylation. These experiments instead indicate that these parameters may be poor therapeutic targets, given that healthy cells display similar attributes to tumour cells. However, many metabolic parameters were statistically different between primary healthy and GBM cells that could illuminate the most promising targets for novel therapeutics (Table 3).

The addition of FCCP to primary GBM cells (Figure 2B) did not elicit an increase in OCR, indicating a loss of reserve capacity. This was also seen in the majority of GBM cell lines (U251MG, U373MG and T98G). One explanation for this is that tumour cells are known to predominantly utilise “the Warburg effect”, reducing their reliance on mitochondrial respiration [4]. Thus, administration of an uncoupling agent may result in a lesser increase in oxygen consumption as the activity of the electron transport chain is already reduced. To support this hypothesis, both the baseline OCR (Figure 1) and the basal mitochondrial rate (Figure 3B) of primary GBM cells were observed as significantly lower than that of primary healthy cells.

Studies often utilise GBM cell lines for metabolic research, due to their relative accessibility and propagation potential. The issue that GBM cell lines differ in their metabolic activity from primary disease cells has been previously examined, yet no previous study has incorporated this number of commonly used GBM cell lines for more comprehensive metabolic profiling. Separate tumour cell lines have been found to respond differently to metabolism-altering compounds, with some eliciting heightened responses, due to their endogenous levels of hypoxia-induced factor 1-alpha [20]. The phosphatase and tensin homolog (PTEN) status of cell lines has been shown to affect metabolic activity [21] as well as p53 [22]. Potentially due to their mutant p53 alleles [23], the primarily glycolytic lines U251 and T98G have been shown to be unable to properly activate AMPK signalling [24] which is critical for survival in a hypoxic environment [25]. In our study, we observed no change in response to rotenone/antimycin A after FCCP administration in T98G cells, potentially due to similar genomic modifications which may be revealed in further study.

There are limitations to this study which must be noted. While several GBM cell lines were examined in this study, this list was not exhaustive. Further, this study utilised the Mito Stress Kit (Seahorse Bioscience, North Billerica, MA, USA) for assessing metabolic parameters, which provided a broad assessment of cellular metabolic activity. Further study into the specifics of glycolytic metabolism, fatty acid oxidation and pentose phosphate pathway metabolism could be illuminated using blocking drugs such as 2-deoxy-D-glucose (2-DG) or 6-aminonicotinamide; these were beyond the scope of this study.

The subgroups of GBM can be classified as proneural, classical and mesenchymal, as determined by Wang, et al. [26], or up to six different subtypes if accounting for DNA methylation, as determined by Brennan, et al. [27]. A further limitation to this study is that the GBM cell lines, and primary cells, have not been transcriptionally or epigenetically profiled to aid in this classification. The clinical data as currently established in the literature for the cell lines used in this paper are listed in Table S1. However, it is worthy to note that these GBM subtypes are driven by different alterations which can affect metabolism, for example Myc [28] or EGFR amplification [29]. The data presented in this study are useful for the recommendation of cell lines to be used as GBM models in metabolic studies. No single cell line appears to reflect the metabolic activity of primary GBM cells completely (Table 3), although it can be deduced that U87MG and T98G cell lines are poor models for mitochondrial metabolism. No cell line accurately reflected the ATP-linked respiration of primary GBM cells. Therefore, cell lines U251MG, U373MG and D54 cells would be most accurate for modelling the mitochondrial metabolism of primary GBM cells, with D54 cells being the preferred choice for studies researching reserve capacity. T98G clearly would be the optimal model for glycolysis-related metabolic research, mirroring all measured attributes of primary GBM cells.

## 4. Materials and Methods

### 4.1. Ethics

For all primary tissue sourced for these experiments, written informed consent was obtained from each subject or from their guardian. Specimens were received from the SA Neurological Tumour Bank (SANTB) which is supported by Flinders University, Flinders Foundation and The Neurosurgical Research Foundation. The ethics for the SANTB is approved by the SAC HREC with approval number 286.10 (received on 10 November 2017).

### 4.2. Cell Line Culture

GBM cell lines (U87MG, U251MG, U373MG, D54 and T98G), were obtained from ATCC (Manassas, VA, USA) and used at passages 6–8. Cells were cultured at 37 °C with 5% CO_2_ in air in DMEM (Sigma-Aldrich, New South Wales, Australia) supplemented with 10% *v*/*v* FBS (Sigma-Aldrich) and 1 mg/mL Antibiotic-Antimycotic (Sigma-Aldrich). Cells were plated at 5 × 10^4^ cells per well in a Seahorse XFe96 Cell Culture Microplate (Seahorse Bioscience; Agilent, Santa Clara, CA, USA), *n* = 5 per cell line with each sample plated in triplicate, before being left at room temperature (RT) for 1 h to reduce the edge effect. Cells were then incubated (24 h, 37 °C) prior to analysis to facilitate cell attachment to the growth surface.

### 4.3. Short Tandem Repeat Profiling

Short tandem repeat (STR) profiling to confirm the genetic identity of each cell line and exclude cross-contamination was performed by the Australian Genome Research Facility (https://www.agrf.org.au/) using the GenePrint 10 assay (Promega, Fitchburg, WI, USA). Genomic DNA for this was extracted from cells using a GenElute mammalian genomic DNA miniprep kit (Sigma-Aldrich). Alleles detected at 10 human loci (THO1, D21S11, D5S818, D13S317, D7S820, D16S539, CSFIPO, AMEL, vWA and TPOX) were compared with the ATCC STR database (https://www.atcc.org/STR_Database.aspx) and using the Cellosaurus STR similarity search tool (https://web.expasy.org/cellosaurus-str-search/) using a match threshold of 80% (results supplied in Table S2).

### 4.4. Primary Brain Cell Culture

Adult human brain tissue was collected at Flinders Medical Centre during neurosurgical resection of glioblastoma tumour. Clinical information on all primary tissue samples can be found in Table S3. A subset of these samples have previously been profiled transcriptionally [30], with MDS analysis stratifying GBM samples as distinct from health tissue controls.

Healthy tissue adjacent to the tumour was obtained (*n* = 3), as well as the glioblastoma tumour tissue (*n* = 3). Each tissue type was collected at the time of resection and placed in 1× phosphate-buffered saline (PBS) solution, (pH 7.4, 4 °C) and transported to the laboratory. The PBS solution was changed within 10 min of resection and the tube was gently inverted for 5 min to clean the tissue.

For some tissues (healthy tissue (*n* = 5), glioblastoma tumour tissue (*n* = 10)), fresh tissue was unavailable due to COVID19 restrictions. For these samples, tissues that had been washed in PBS and cryopreserved within 15 min of resection were plated in a 6-well plate with 2 mL DMEM (Sigma-Aldrich) supplemented with 10% *v*/*v* FBS (Sigma-Aldrich) and 1 mg/mL Antibiotic-Antimycotic (Sigma-Aldrich) in a 37 °C incubator with 5% CO_2_ in air for 1 h. After 1 h, this media was replaced for a further 1 h. This process was then repeated once more.

Fat was removed and the tissue was cut into 2 mm^3^ pieces. The healthy tissue was transferred into a chelation buffer (3 mM ethylenediaminetetraacetic acid (EDTA), 50 µM Dithiothreitol (DTT) in PBS) and the tumour tissue was transferred into Accumax™ (Thermo-Fisher Scientific, Waltham, MA, USA) and incubated at RT for 1 h (with gentle rocking). The tissue was then placed in PBS (5 mL, 4 °C) and vigorously agitated for 5 s. Supernatant was collected and this process was repeated until the supernatant remained clear after agitation. Collected supernatant was centrifuged (400× *g*, 3 min, RT) and then supernatant was removed. The pellet was resuspended in 1.5 mL of ice-cold PBS before being passed through a cell sieve (70 µm) to remove debris. Cells were then centrifuged again at 400× *g* for 1 min at room temperature before resuspension in 1 mL growth medium (as above) and cells were counted using a hemocytometer and filtered trypan blue (1%; Sigma Aldrich) to exclude dead cells. Cells were then plated at 5 × 10^4^ cells per well in a Seahorse XFe96 Cell Culture Microplate (Seahorse Bioscience, Agilent), in triplicate wells for *n* = 13 tumours, *n* = 8 normal, before being left at RT for 1 h to minimize edging. Cells were then moved into the 37 °C incubator (20% O_2_, 5% CO_2_) for 2 h to allow for further adhesion.

To maintain a mixed population of neural cells (including astrocytes, oligodendrocytes and neurons), cells were grown in a media containing serum for only 3 h. This time frame was chosen as it is not long enough to significantly diminish neuron growth whilst also encouraging astrocyte growth which is retarded in serum-free media [31,32].

### 4.5. Metabolic Flux Analysis

To assess flux through cytoplasmic and mitochondrial metabolic pathways, cells were assayed using a Seahorse Bioscience XF Analyser and Mitostress test kit according to the manufacturer’s instructions (Agilent Technologies (Santa Clara, CA, USA) User Guide Kit 103015-100). Pharmacological compounds were given at a final well concentration of oligomycin (1.5 μM), FCCP (1.0 μM) and rotenone/antimycin A (0.5 μM). Equilibration of the sensor cartridge was performed as per the manufacturer’s recommendations. Three measurement cycles at five-minute intervals were performed prior to the first and following every compound injection as is standard practice for metabolic flux analysis. Cells from fresh tissue compared with frozen tissue was assessed to ensure no effect of cryopreservation on metabolism (shown in Figure S1).

When the assays were complete, plates were fixed with 4% paraformaldehyde before imaging (Cytation 5; BioTek, Winooski, VT, USA) with high-resolution brightfield. This calculated the number of cells per well which was used for normalisation between wells in each plate.

### 4.6. Metabolic Capacity Calculations

**Figure 5:**
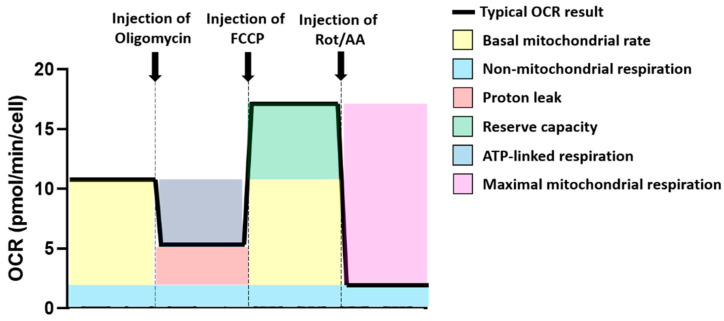
Use of the Oxygen Consumption Rate (OCR) profile to calculate mitochondrial and non-mitochondrial-related metabolic and oxygen consumption parameters. Different colours have been used to represent the different OCR parameters that have been calculated for these experiments. (yellow) represents the basal mitochondrial rate; (blue) represents non-mitochondrial respiration; (red) indicates proton leak; (green) represents reserve capacity; (navy) represents ATP-linked respiration; (purple) indicates maximal mitochondrial respiration. A bolded black line shows the typical OCR profile. A dashed grey line indicated compound injection points.

The Oxygen Consumption Rate (OCR) was used for calculation of mitochondrial and related metabolic parameters of the cells tested (Figure 5). Non-mitochondrial respiration was assessed based on OCR after rotenone/antimycin A injection. The basal mitochondrial rate was calculated as OCR at baseline, minus non-mitochondrial respiration rate. Proton leak was calculated as OCR after oligomycin injection, minus non-mitochondrial respiration rate. ATP-linked respiration was calculated as baseline OCR, minus OCR after oligomycin injection. Reserve capacity was calculated as OCR after injection with FCCP, minus baseline OCR. Finally, maximal mitochondrial respiration was calculated as OCR after FCCP injection, minus non-mitochondrial respiration.

**Figure 6:**
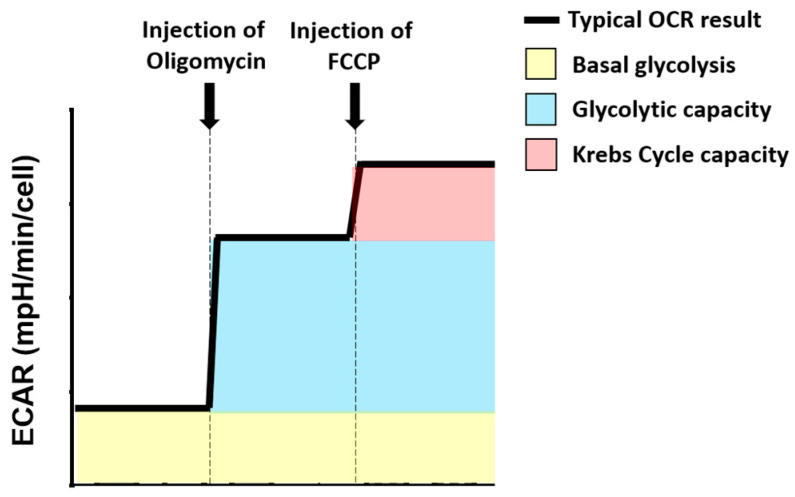
Use of the Extracellular Acidification Rate (ECAR) profile to calculate glycolysis-related metabolic parameters. Different colours have been used to represent the different ECAR parameters that have been calculated for these experiments. (yellow) represents basal glycolytic rate; (blue) represents glycolytic capacity; (red) indicates Krebs cycle capacity. A bolded black line shows the typical OCR profile. A dashed grey line indicated compound injection points.

The Extracellular Acidification Rate (ECAR) of cells was used for calculation of glycolytic metabolic parameters (Figure 6). Basal glycolysis was based on the ECAR at baseline. Glycolytic capacity was calculated as the ECAR after oligomycin injection, minus the basal ECAR. Krebs cycle capacity was calculated as the ECAR after FCCP injection, minus glycolytic capacity.

### 4.7. Statistics

Statistical analysis was undertaken using Prism 7 (GraphPad). Data were assessed for normality distribution and a one-way ANOVA (non-parametric) test was conducted using a Kruskal–Wallis multiple comparisons test. Differences between groups were considered significant for *p* ≤ 0.05. All tests of statistical significance are two-sided.

## 5. Conclusions

Whenever possible, primary human GBM cells should be sourced for metabolism studies as they will provide the most accurate representation of metabolic responses. However, GBM cell lines can be useful substitutes for measuring specific metabolic responses and therapeutics targeting specific components of these pathways. We recommend the use of U251MG, U373MG and D54 cells for mitochondrial metabolism studies, and T98G cells for glycolysis metabolism studies.

## Figures and Tables

**Table 1:** Values of significance for cell comparisons of OCR-related metabolic parameters.

Comparison	Parameter	Healthy	1′ GBM	U87MG	U251MG	U373MG	D54	T98G
Compared to 1′ Healthy Cells	Non-mito respiration	6.57 ± 0.21	6.11 ± 0.19	*p* = 0.007 15.95 ± 0.76	2.82 ± 0.21	4.79 ± 0.62	*p* < 0.001 0.51 ± 0.22	*p* = 0.012 15.61 ± 0.89
	Basal mito rate	5.30 ± 0.28	*p* = 0.043 2.76 ± 0.19	*p* < 0.001 0.02 ± 0.01	*p* = 0.003 2.00 ± 0.33	2.82 ± 0.31	3.92 ± 0.24	*p* < 0.001 0.05 ± 0.25
	ATP-linked resp rate	4.95 ± 0.36	*p* < 0.001 1.86 ± 0.16	1.33 ± 0.51	4.36 ± 0.36	5.18 ± 0.99	4.41 ± 0.62	6.11 ± 0.27
	Proton leak	0.07 ± 0.23	0.78 ± 0.13	*p* = 0.006 0.00 ± 0.00	2.34 ± 0.21	2.14 ± 0.36	0.04 ± 0.22	*p* = 0.006 0.01 ± 0.00
	Reserve capacity	4.74 ± 0.17	*p* = 0.037 10.17 ± 0.29	5.22 ± 0.43	3.51 ± 0.49	0.04 ± 0.46	7.10 ± 0.48	0.15 ± 0.43
	Maximal mito resp rate	9.80 ± 0.24	12.99 ± 0.38	11.99 ± 0.56	9.15 ± 0.41	9.48 ± 0.38	12.59 ± 0.46	*p* < 0.001 0.00 ± 0.01
Compared to 1′ GBM	Non-mito respiration	-	-	*p* = 0.004	-	-	*p* = 0.002	*p* = 0.007
	Basal mito rate	*p* = 0.043	-	*p* = 0.002	-	-	-	*p* = 0.020
	ATP-linked resp rate	*p* < 0.001	-	*p* = 0.004	*p* = 0.004	*p* < 0.001	*p* < 0.001	*p* < 0.001
	Proton leak	-	-	*p* = 0.011	-	-	-	*p* = 0.011
	Reserve capacity	*p* = 0.037	-	-	*p* < 0.001	*p* < 0.001	-	*p* < 0.001
	Maximal resp rate	-	-	-	-	-	-	*p* < 0.001

Where significant (*p* < 0.05), *p*-values are shown. Data presented represent the mean ± SEM.

**Table 2:** Values of significance for cell comparisons of Extracellular Acidification Rate (ECAR)-related metabolic parameters.

Comparison	Parameter	Healthy	1′ GBM	U87MG	U251MG	U373MG	D54	T98G
Compared to 1′ Healthy Cells	Basal glycolysis	4.29 ± 0.30	4.05 ± 0.35	*p* = 0.006 0.16 ± 0.36	*p* = 0.006 0.31 ± 0.34	*p* = 0.009 2.07 ± 0.23	*p* = 0.009 0.06 ± 0.26	5.19 ± 0.27
	Glycolytic capacity	3.30 ± 0.22	4.73 ± 0.29	*p* = 0.038 1.00 ± 0.32	3.02 ± 0.22	*p* < 0.001 0.84 ± 0.18	*p* < 0.001 0.00 ± 0.28	4.02 ± 0.20
	Krebs cycle capacity	1.01 ± 0.22	*p* = 0.002 3.63 ± 0.13	0.56 ± 0.21	*p =* 0.002 0.00 ± 0.03	0.92 ± 0.20	0.01±0.26	2.14 ± 0.17
Compared to 1′ GBM	Basal glycolysis	-	-	*p* = 0.004	*p* < 0.001	*p* = 0.025	*P* < 0.001	-
	Glycolytic capacity	-	-	*p* = 0.035	-	*p* < 0.001	*p* < 0.001	-
	Krebs cycle capacity	*p* = 0.002	-	*p* = 0.005	*p* < 0.001	*p* = 0.001	*p* < 0.001	-

Where significant (*p* < 0.05), *p*-values are shown. Data presented represent the mean ± SEM.

**Table 3:** Which cells reflect the metabolic activity of primary GBM cells?

Category	Parameter	Healthy	U87MG	U251MG	U373MG	D54	T98G
OCR related	Non-mito respiration	✓	-	✓	✓	-	-
	Basal mito rate	-	-	✓	✓	✓	-
	ATP-linked resp rate	-	-	-	-	-	-
	Proton leak	✓	-	✓	✓	✓	-
	Reserve capacity	-	✓	-	-	✓	-
	Maximal mito resp rate	✓	✓	✓	✓	✓	-
ECAR related	Basal glycolysis	✓	-	-	-	-	✓
	Glycolytic capacity	✓	-	✓	-	-	✓
	Krebs cycle capacity	-	-	-	-	-	✓

The metabolic activity of each cell type (primary healthy cells, U87MG, U251MG, U373MG, D54, T98G) has been compared with the metabolic activity of primary GBM cells for each metabolic parameter assessed. A tick (✓) has been placed in the corresponding box if the cell reflects the metabolic activity of primary GBM cells for the parameter, as determined using absolute values and median of cells compared with primary GBM.

## Supplementary Materials

The following are available online at https://www.mdpi.com/2072-6694/12/12/3722/s1, Table S1: Clinical data for GBM cell lines; Table S2: Short tandem repeat analysis of GBM cell lines; Table S3: Clinical data for primary tissue; Figure S1: Comparison of OCR-related and ECAR-related metabolic parameters between fresh and frozen tissue of primary neural and GBM cells.
