# Supplementary material for: The Suitability of Glioblastoma Cell Lines as Models for Primary Glioblastoma Cell Metabolism
Source: Cancers (Basel). 2020 Dec 11;12(12):3722. doi: 10.3390/cancers12123722 (PMC7764800; doi:10.3390/cancers12123722)
Supplement: Supplementary file 1 [file cancers-12-03722-s001.pdf]

cancers

Supplementary Material: The Suitability of Glioblastoma Cell Lines as Models for Primary Glioblastoma Cell Metabolism

Anya L. Arthurs, Damien J. Keating, Brett W. Stringer and Simon J. Conn

Table S1. Clinical data for GBM cell lines.

Clinical Data	D54	T98G	U251	U373	U87
Category	Glioblastoma	Glioblastoma	Glioblastoma	Glioblastoma	Glioblastoma
IDH1	-	WT	WT	-	WT
TERT	-	-	C228T	C228T	C228T
TP53	-	p.Met237Ile	p.Arg273His hom	p.Arg273His hom	-
PTEN	hom del	p.Leu42Arg	p.Glu242Valfs*15	p.Glu242Valfs*15	c.209 + 1G > T hom
NF1	-	-	-	-	p.Phe1247Ilefs*18 (het)
Origin	53 year old male	61 year old male	75 year old male	75 year old male	male, age unknown

Abbreviations: WT = wildtype; C228T = telomerase reverse transcriptase gene *(TERT)* promoter mutation where the cytosine residue at position 228 is replaced with thymine; p.Met237Ile = a sequence change which replaces methionine with isoleucine at codon 237 of the tumour protein P53 (TP53) protein; p.Arg273His hom = a sequence change which replaces arginine with histidine at codon 273 of the TP53 protein (homozygous); p.Leu42Arg = a sequence change which replaces leucine with arginine at codon 42 of the Phosphatase and tensin homolog (PTEN) protein; p.Glu242Valfs*15 = a sequence change which replaces glutamine with valine at codon 242 of the PTEN protein and results in a frameshift that creates a premature STOP codon; c.209 + 1G > T hom = ; p.Phe1247Ilefs*18 (het) = a nucleotide insertion that replaces a phenylalanine with an isoleucine at codon 1247and causes a frameshift resulting in a premature STOP codon (heterozygous).

Table S2. Short tandem repeat analysis of GBM cell lines.

Cell Line	AMEL	CSF1PO	D13S317	D16S539	D21S11	D5S818	D7S820	TH01	TPOX	vWA
DT54	X	9,12	11	12	28,32.2	11,12	11	6,9.3	8,11	16,20
T98G	X,Y	10,12	13	13	28,32.2	10,12	9,10	7,9.3	8	17,20
U251	X	11,12	10,11	12	29	11,12	10,12	9.3	8	16,18
U373	X,Y	11,12	10,11	12	29,30	11,12	10,12	9.3	8	16,18
U87	X,Y	10,11	8,11	12	28,32.2	11,12	8,9	9.3	8	15,17

X and Y indicate the X and Y chromosomes. Numbers represent the alleles at each locus.

Table S3. Clinical data for primary tissue.

Sample ID	Tumour Type	Grade
57	Glioblastoma	IV
61	Glioblastoma	IV
169	Glioblastoma	IV
134	Glioblastoma	IV
160	Glioblastoma	IV
170	Glioblastoma	IV
199	Glioblastoma	IV
200	Glioblastoma	IV
203	Glioblastoma	IV
240	Glioblastoma	IV
255	Glioblastoma	IV
322	Glioblastoma	IV
362	Glioblastoma	IV
61	Matched Control	Healthy

149	Control	Healthy
170	Matched Control	Healthy
204	Control	Healthy
216	Control	Healthy
236	Control	Healthy
239	Control	Healthy
261	Control	Healthy

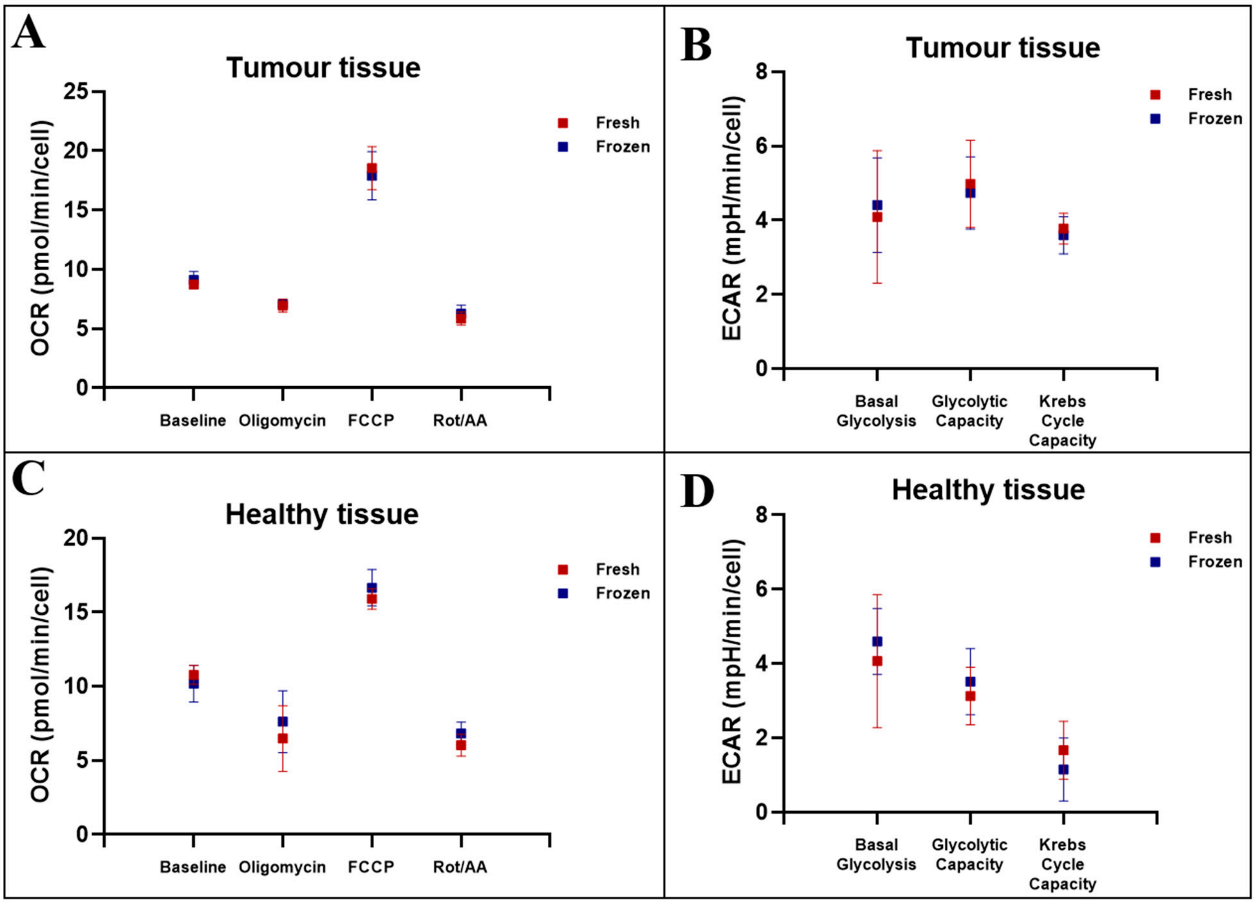

Figure S1. Comparison of OCR-related and ECAR-related metabolic parameters between fresh and frozen tissue of primary neural and GBM cells. (**A**) OCR-related metabolic parameters of GBM tumour tissue; (**B**) ECAR-related metabolic parameters of GBM tumour tissue; (**C**) OCR-related metabolic parameters of healthy neural tissue; (**D**) ECR-related metabolic parameters of healthy neural tissue. For all data points in fresh primary healthy tissue n=3, in frozen primary healthy tissue $n = 5$, in fresh GBM tumour tissue $n = 3$, in frozen GBM tumour tissue $n = 10$; plated in triplicate. Data are represented as mean ± SEM, red indicates fresh tissue samples, blue indicates frozen tissue samples. There were no significantly different changes in metabolic parameters between any fresh and frozen groups, where statistical significance is assessed as $p \leq 0.05$.

Publisher's Note: MDPI stays neutral with regard to jurisdictional claims in published maps and institutional affiliations.

© 2020 by the authors. Licensee MDPI, Basel, Switzerland. This article is an open access article distributed under the terms and conditions of the Creative Commons Attribution (CC BY) license (http://creativecommons.org/licenses/by/4.0/).
